# The False Economy of Seeking to Eliminate Delayed Transfers of Care: Some Lessons from Queueing Theory

**DOI:** 10.1007/s40258-022-00777-2

**Published:** 2022-12-18

**Authors:** Richard M. Wood, Alison L. Harper, Zehra Onen-Dumlu, Paul G. Forte, Martin Pitt, Christos Vasilakis

**Affiliations:** 1 UK National Health Service (BNSSG ICB), NHS Bristol, North Somerset and South Gloucestershire Integrated Care Board, 360 Bristol, Marlborough St, Bristol, BS1 3NX UK; 2 School of Management, University of Bath, Bath, UK; 3 Medical School, University of Exeter, Exeter, UK; 4 Health Data Research UK, South West Better Care Partnership, Bristol, UK

## Abstract

**Background:**

It is a stated ambition of many healthcare systems to eliminate delayed transfers of care (DTOCs) between acute and step-down community services.

**Objective:**

This study aims to demonstrate how, counter to intuition, pursual of such a policy is likely to be uneconomical, as it would require large amounts of community capacity to accommodate even the rarest of demand peaks, leaving much capacity unused for much of the time.

**Methods:**

Some standard results from queueing theory—a mathematical discipline for considering the dynamics of queues and queueing systems—are used to provide a model of patient flow from the acute to community setting. While queueing models have a track record of application in healthcare, they have not before been used to address this question.

**Results:**

Results show that ‘eliminating’ DTOCs is a false economy: the additional community costs required are greater than the possible acute cost saving. While a substantial proportion of DTOCs can be attributed to inefficient use of resources, the remainder can be considered economically essential to ensuring cost-efficient service operation. For England’s National Health Service (NHS), our modelling estimates annual cost savings of £117m if DTOCs are reduced to the 12% of current levels that can be regarded as economically essential.

**Conclusion:**

This study discourages the use of ‘zero DTOC’ targets and instead supports an assessment based on the specific characteristics of the healthcare system considered.

**Supplementary Information:**

The online version contains supplementary material available at 10.1007/s40258-022-00777-2.

## Key Points for Decision Makers

**Table:** 

It is a stated aim of many healthcare systems to ‘eliminate’ delays in transfer of care between acute and community services.
Mathematical modelling suggests that this requires uneconomically large amounts of downstream community capacity, the cost of which outweighs the acute cost saving.
For England’s National Health Service (NHS), substantial cost savings are possible if delays are reduced to the 12% that can be considered economically essential.

## Introduction

In a nutshell, a delayed transfer of care (DTOC) occurs when a patient is ready for discharge from hospital but is still occupying a bed [1]. This can happen for a variety of reasons, such as disputes or patient choice, but is typically due to insufficient downstream capacity for patients requiring some form of step-down or continuing non-acute care [2–4].

In England’s National Health Service (NHS), 1.14m acute bed days were lost due to DTOCs in the 12-month period before the coronavirus disease 2019 (COVID-19) pandemic was declared in March 2020 [5]. Some of these (28%) were attributable to a lack of provision of local authority-funded social care, while approximately half (48%) were related to the unavailability of short-term step-down care (otherwise known as intermediate care) provided by NHS-funded community services [1].

At first glance, it would appear that DTOCs are wholly negative and should be reduced to zero within well-performing healthcare systems, and this is the impression given by many NHS organisations. Manchester and Surrey talk of plans to “eliminate DTOCs” [6, 7]. A major London healthcare system claims that hospitals have “accepted their challenge to eliminate DTOCs” [8] and, in light of COVID-19, the Isle of Wight system states “it has never been more important to eliminate DTOCs” [9]. Meanwhile, in Scotland, the Health Secretary has pledged to “eradicate bed blocking” [10]. Mention of ‘eliminate’ and ‘eradicate’ in relation to DTOCs also extends to the academic literature [11, 12]. However, should hospitals and healthcare systems really seek to eliminate DTOCs?

Eliminating DTOCs caused by insufficient downstream community capacity is entirely possible if the arrivals (patients becoming ready for discharge from the acutes) and lengths of stay (in the community service) are perfectly constant. For example, if we have exactly five acute patients becoming ready for discharge every day to bed-based community care (community hospitals or in ‘short-stay’ care home beds) and each patient has a community length of stay (LOS) of exactly 12 days, then 60 beds’ worth of community capacity would be sufficient. With this amount of capacity, there would never be any ‘queue’ at the acute hospitals (i.e. zero DTOCs) and all community capacity would be fully utilised all of the time.

**Fig. 1:**
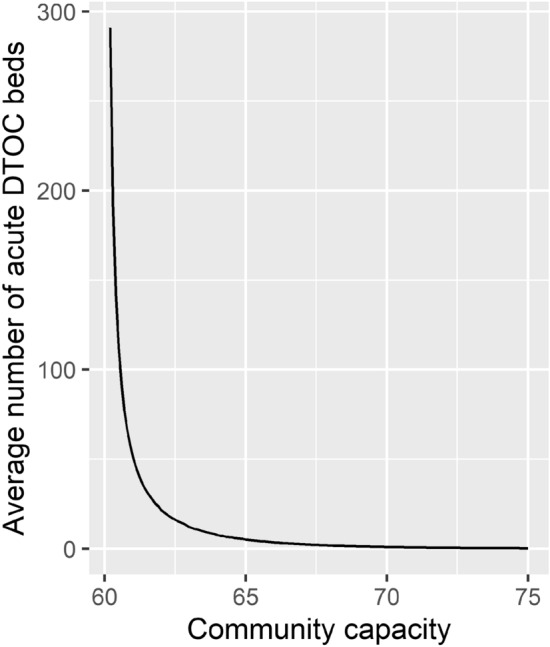
Effect of post-acute community capacity on the average number of acute beds subject to DTOCs, assuming a mean of five acute patients becoming ready for community discharge each day and a mean community LOS of 12 days. Modelling with some typical variability around arrivals (assumed Poisson) and LOS (assumed exponential) shows that the ‘averages-based’ community capacity (5×12) to treat 60 concurrent patients is insufficient, with large acute DTOCs developing. Results are produced using the method detailed in the Methods section. *DTOCs* delayed transfers of care, *LOS* length of stay

However, we know from Queueing Theory, a mathematical subdiscipline of Operational Research concerning the dynamics of queues, that when there is variability in the arrival rate and/or LOS then some amount of queueing is inevitable given the various peaks and troughs that will occur over time, such as having a number of arrivals in a short time period or having a group of long-staying patients admitted all at once [13, 14]. To prevent a significant queue forming then extra ‘slack’ is required in the system, and this can be achieved through increasing community bed capacity. The amount of additional capacity required depends on the amount of queueing considered tolerable (Fig. 1).

While able to reduce costly queueing due to DTOCs, this extra downstream capacity comes at a cost itself in terms of the additional community beds, domiciliary carers, and other support staff required. The question is, at what point do these additional costs start to outweigh the associated savings in reducing acute DTOCs? If this is known, and appropriate (non-zero) DTOC targets are set, then healthcare systems can work towards community discharge pathways of greater cost optimality.

The aim of this study was to illustrate the false economy of seeking to eliminate DTOCs and to show that some amount of ‘bed blockage’ is beneficial to reducing the total costs across the continuum of care. This is approached through applying established Queueing Theory formulae to a major NHS healthcare system in South West England to calculate the optimal amount of acute DTOCs and community capacity required. Sensitivity to a range of parameters is considered, including the acute- community cost ratio and level of variability in community LOS, as well as the size of the healthcare system. Cost savings for England are also estimated if the optimal amount of DTOCs is to be achieved.

It should be noted that this study considers only the financial costs related to the part of the patient pathway of interest, i.e. the costs of caring for acute patients experiencing DTOCs and of operating the downstream community service. By not taking into account the wider costs potentially associated with patient ‘deconditioning’ (i.e. those related to additional care needs), the analysis presented here essentially assumes that patient care (beyond medical fitness for discharge) and outcomes are not impacted by the care setting. The possible effects of this assumption are considered further in the Discussion section. Note also that only ‘step-down’ care is considered in this study (the healthcare systems of some countries may also include ‘step-up’ care for admission avoidance purposes).

## Methods

### Queueing Theory

To address the aims of this study, it is necessary to introduce some terminology from Queueing Theory. First, we have *arrivals*. In our case, these are patients becoming ready for discharge from the acute hospital and requiring continuing care in the community setting. As is typically assumed for healthcare models, the arrival of one patient can be considered independent of the arrival of another [15]. Thus, the number of arrivals occurring in an interval of time can be described by a Poisson distribution, meaning the time between successive arrivals is exponentially distributed, or *Markovian*. Second, we have *service times*, or LOS. In our case, this represents the durations of time for which patients are under the care of the community service provider (starting from when they are transferred from the acute hospital), and is subject to some degree of variability. Third, we have *capacity*, which represents the maximum number of patients that can be cared for concurrently by the community service provider (either at home through care visits or in a bed-based unit). In Queueing Theory, Kendall’s Notation is used to summarise these three properties [16]. In our case, we have an *M|G|c* queue, i.e. Markovian arrivals, some sort of general service time (LOS) distribution, and a capacity for *c* patients.

The formulae needed for the *M | G | c* queue is actually based on derivations for the *M | M | c* queue (i.e. the same queue but assuming that LOS is exponentially distributed, or *Markovian*). For this type of queue, Erlang’s C Formula, an established result in Queueing Theory [17], gives the probability of an arriving patient finding no available community capacity, and thus experiencing a DTOC (Eq. 1):

1 $$\overline{C }={\left[1+\left(1-\rho \right)\left(\frac{c!}{{\left(c\rho \right)}^{c}}\right)\sum_{k=0}^{c-1}\frac{{\left(c\rho \right)}^{k}}{k!}\right]}^{-1}$$

Here, $\rho$ is the *traffic intensity*, which measures the ratio of demand to capacity. This is calculated through dividing the mean of the Poisson arrival rate, $\lambda$, by the mean service rate, $c\mu$, where $\frac{1}{\mu }$ is the mean of the general service time distribution representing community LOS. The smaller $\rho$ is, the less pressure the service will be under, and thus the less queueing there will be.

The average waiting time, $\overline{W }$, for an *M | M | c* queue is calculated [18] as (Eq. 2):

2 $$\overline{W }=\frac{\overline{C} }{c\mu -\lambda }$$

This is related to the *M | G | c* queue through the approximation (Eq. 3) [19]:

3 $$W\approx \frac{{V}^{2}+1}{2}\overline{W }.$$

Here, *V* measures the amount of dispersion in the LOS distribution (specifically, its coefficient of variation, calculated by its standard deviation over its mean).

Average queue length, here representing the number of acute DTOC beds (i.e. those occupied at any time due to unavailable downstream capacity), can be calculated through the average waiting time by Little’s Law (Eq. 4) [20]:

4 $$L=\lambda W.$$

Therefore, if an acute bed costs $\alpha$ per day, then the average daily cost of DTOCs borne in the acute setting is $\alpha L$. If the cost ratio of community to acute care is $\beta$ (presumed less than one), such that the cost of community care is $\beta \alpha$ per provided-for patient per day, then the corresponding daily cost borne in the community setting is $\beta \alpha c$. The total daily cost is thus calculated as the addition of the variable cost of acute discharge delays and the fixed cost of procured community capacity (Eq. 5):

5 $$T=\alpha L+\beta \alpha c.$$

It should be noted that the value of this equation does not depend on the exact values of $\lambda$ and $\mu$, but their ratio as expressed in $\rho$ (recalling $\rho =\lambda /c\mu$), as well as the coefficient of variation of LOS, $V$. This can be shown by combining Eqs. 1–5 to yield (Eq. 6):

6 $$T=\alpha \frac{{V}^{2}+1}{2}\frac{\rho }{1-\rho }{\left[1+\left(1-\rho \right)\left(\frac{c!}{{\left(c\rho \right)}^{c}}\right)\sum_{k=0}^{c-1}\frac{{\left(c\rho \right)}^{k}}{k!}\right]}^{-1}+\beta \alpha c$$

As can be seen, the value of *T* depends only on $\alpha$, $\beta$, *V*, *c*, and $\rho$, and not λ and μ directly. In other words, the total cost does not depend on the exact mean arrival and service rate values (only their ratio).

### Data and Calibration

We apply the above formulae to a major healthcare system located in and around Bristol, in the south west of England. The Bristol, North Somerset and South Gloucestershire (BNSSG) healthcare system serves a 1 million resident population across a mixture of large metropolitan areas and rural and coastal locations [21]. The large metropolitan area of Bristol contains a higher proportion of younger individuals and has a more culturally and ethnically diverse demographic [22]. Rural and coastal areas contain a greater proportion of older individuals and pockets of severe deprivation [23]. Overall, the age profile of BNSSG is similar to that of England [21].

The cost of an acute bed day ($\alpha$) is estimated at £346, based on the latest available national reference cost data that contains such a metric, from the financial year 2017/2018 [24]. From local data from the financial year 2020/2021, we estimated an average daily community cost across both NHS-funded home-based and bed-based care of £138 (i.e. $\beta$ = 138/346 = 0.399). Using these data, we also estimated the coefficient of variation of community service LOS (*V*) of 0.965 (thus indicating slightly less dispersion than the commonly used exponential distribution, which has *V* = 1). Community service capacity (*c*) was estimated at 531, noting that this should be considered a relatively large service provider, given the size of the population covered. Finally, traffic intensity ($\rho$) was estimated at 0.98, which suggests a very busy service (also supported by the data and the high levels of community service occupancy observed). For more details on how the parameter estimates were obtained, see the electronic supplementary material (ESM).

## Results

### Optimal Amount of Acute Delayed Transfers of Care

**Fig. 2:**
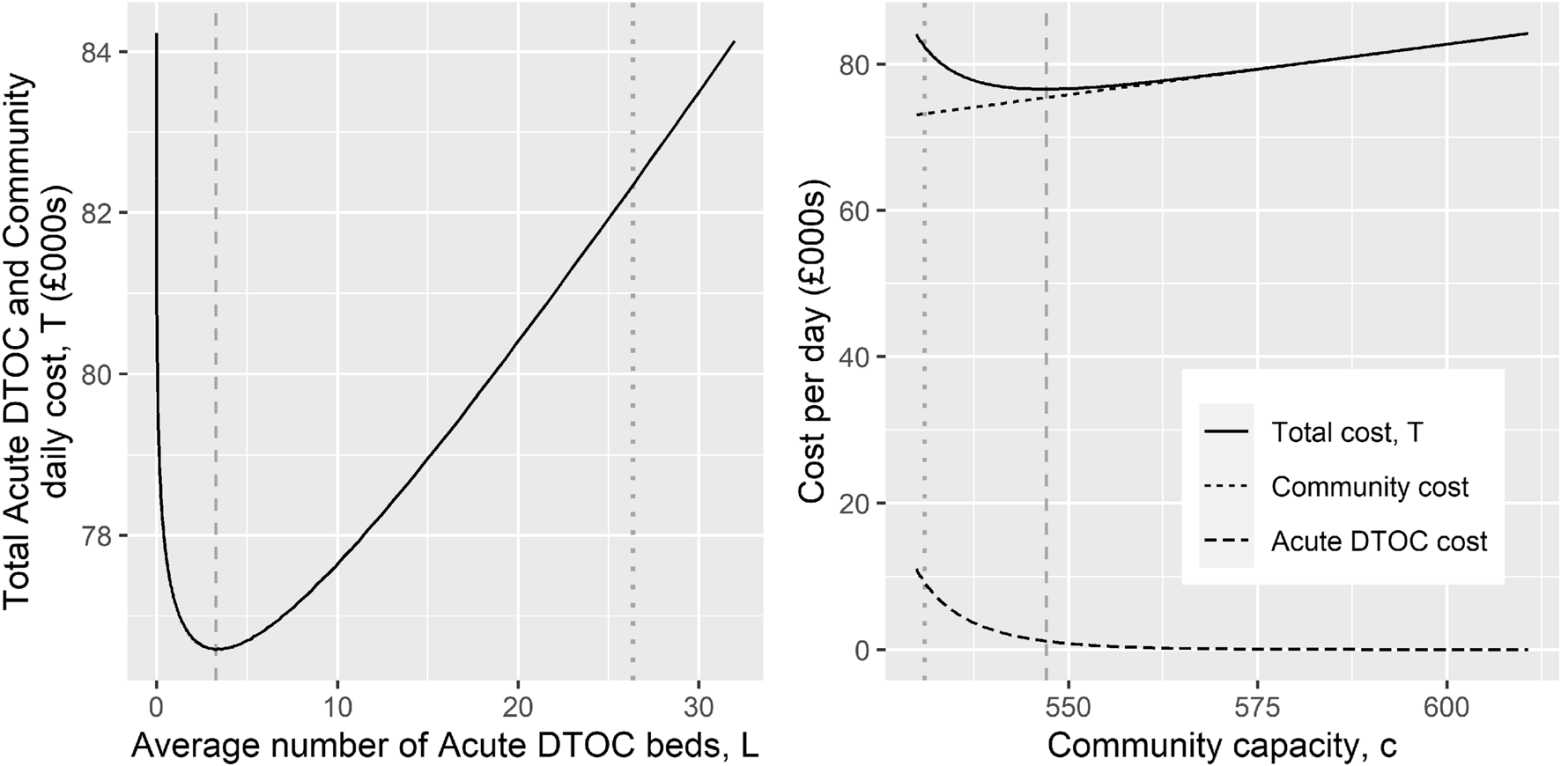
Modelled results showing the optimal amount of acute DTOCs (left-hand side) and community capacity (right-hand side) required to minimise total acute and community cost. Optimal levels are represented by the dashed grey vertical lines, with the dotted grey vertical lines representing the actual levels at the healthcare system studied. *DTOCs* delayed transfers of care

With the Queueing Theory formulae calibrated to the local healthcare system, we can examine the main aim of this study: highlighting the economic benefit for some amount of DTOCs, or ‘bed blocking’ in acute care. In varying the hypothetical levels of NHS-funded, time-limited ‘step-down’ community care capacity, *c*, within Eqs. 4 and 6, we can plot the relationship between acute DTOCs and total costs (Fig. 2, left-hand side). This clearly shows that the minimum total cost is achieved when operating at an above-zero amount of average acute DTOCs.

Moreover, it shows that costs increase sharply when average acute DTOCs are lessened to the point of elimination. This is being driven by the large amounts of downstream community capacity required to achieve such low levels of queueing. Conversely, if there is insufficient community capacity, then acute DTOC costs increase rapidly and non-linearly, making a significant contribution to the total cost (Fig. 2, right-hand side). Figure 2 also indicates that the studied healthcare system could reduce costs by increasing community capacity by 3%, from 531 to the theoretical optimal of 547. This would reduce the average number of acute DTOC beds from 26.3 to 3.3 and realise a 7% saving in daily operating costs.

### Effect of Acute-Community Cost Ratio and Variability in Length of Stay

**Fig. 3:**
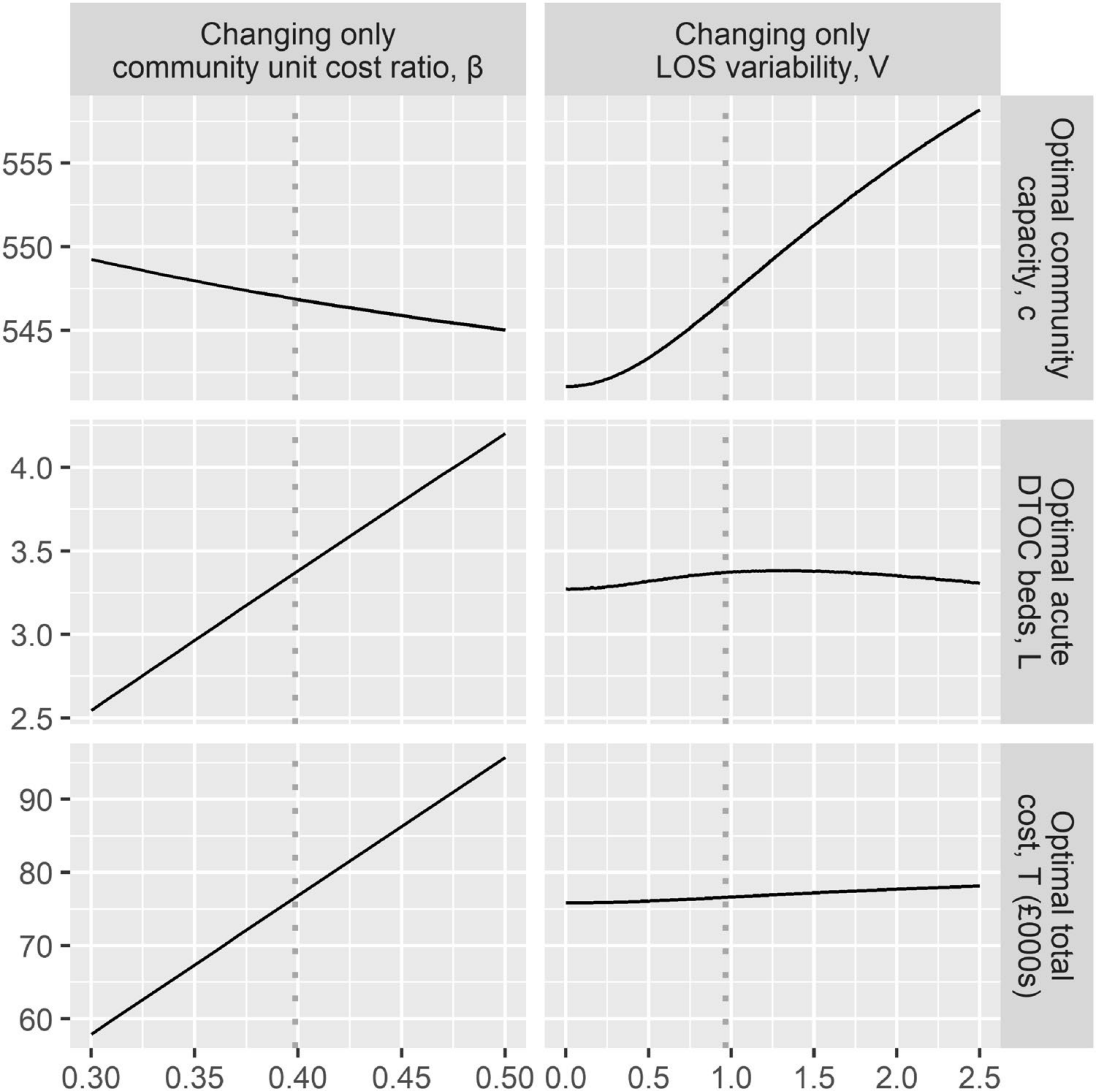
Modelled results showing the effect of changing only the community cost ratio (left-hand side) or the amount of LOS variability (right-hand side) on optimal levels of community capacity, acute DTOCs, and optimal cost. The dotted grey vertical lines represent the actual levels of community cost ratio and LOS variability at the healthcare system studied. *DTOCs* delayed transfers of care, *LOS* length of stay

Changing the ratio, $\beta$, of community to acute unit costs can have a profound effect on optimal resource allocation and utilisation (Fig. 3, left-hand side). As community unit costs increase, then a greater premium is put on community capacity, meaning that it becomes more cost effective to accept a larger amount of acute DTOCs. Essentially, as the cost gap narrows, community capacity is penalised in favour of more ‘bed blocking’. Here, if the community cost ratio is increased from 0.4 to half that of an acute bed, then achieving cost optimality means trading away capacity to care for two patients in the community for an extra one acute bed required to support the greater level of discharge delays.

Another aspect that can affect resource allocation and utilisation is the amount of variability in community LOS, referred to previously by *V* (Fig. 3, right-hand side). When there is zero variability in LOS (i.e. every patient stays exactly the same length of time in community care) then less community capacity is required, and acute DTOCs and total costs also reduce slightly (note that some acute DTOCs still occur given variability in the arrival rate). When LOS variability increases, then so too does the optimal capacity requirement due to the greater volume of potential ‘shocks’ that need to be absorbed (i.e. longer patient stays). If these are not absorbed through additional community capacity, then they are manifest in the more costly acute setting in the form of DTOCs. While the total cost differences appear small on the plotted scale, they can provide considerable annual savings. For instance, if through more targeted efforts to reduce longer community stays, LOS variability could be reduced from 0.965 (dotted grey vertical line) to 0.5 (bottom-right panel), then this would account for a £510 daily saving, totalling £186k annually.

### Effect of Healthcare System Size

Up to this point, all modelling has been based on a particular ‘arrival rate’ of acute patients becoming ready for discharge to the community setting; specifically, that arrival rate of the relatively large healthcare system considered here. Yet, what amounts of acute DTOCs should smaller or even larger healthcare systems work to? Smaller healthcare systems (essentially by definition) would have a lower throughput (arrival rate) and larger healthcare systems a higher throughput. The premise being tested is whether the optimal amount of acute DTOCs scales linearly with size, or whether there is, as would reasonably be expected, an element of queueing ‘economies of scale’.

**Fig. 4:**
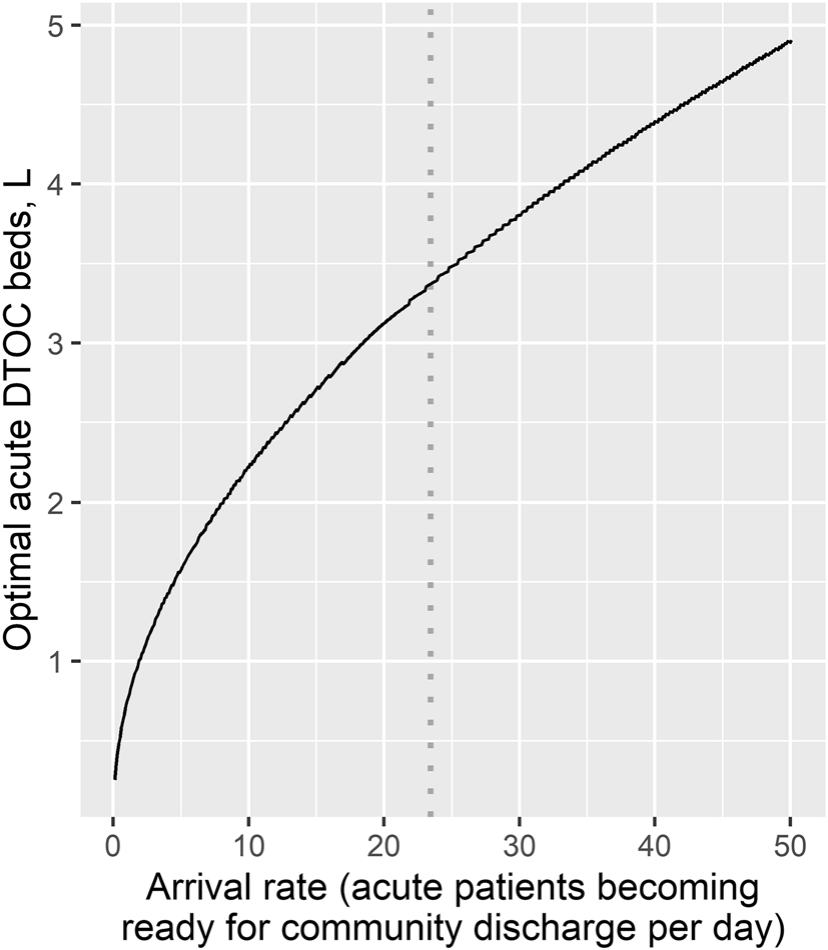
Modelled results showing the optimal amount of acute DTOC beds for different sized healthcare systems, as represented through the ‘arrival’ rate of acute patients requiring community care upon discharge. The dotted grey vertical line represents the arrival rate at the healthcare system studied. *DTOCs* delayed transfers of care

Modelled results show that the relationship is non-linear (Fig. 4). For the first ten daily patient arrivals, an average 2.2 ‘blocked’ acute beds should be accepted. Yet, for the second ten daily arrivals, only an extra 0.9 acute beds should be tolerated, and fewer still (0.7) for the next set of ten. Eventually, the advantages of this queueing ‘economy of scale’ settles down, and the relationship becomes linear for progressively larger systems (Fig. 4). Essentially, this reveals that smaller healthcare systems need to accept proportionately more acute DTOCs to be cost-optimal than larger healthcare systems.

### Cost Saving Potential for England

It was earlier estimated that for the studied 1 million population healthcare system, a saving of 7% could be made on total costs were pathway resources appropriately rebalanced. The 7% saving would represent £5725 per day, totalling £2.1m annually. Key to the specific value of this saving is the typical number of acute beds being occupied by DTOC patients awaiting transfer to a community care setting. This is 26.3 for the healthcare system studied but, were it closer to the optimal 3.3 acute beds, then clearly there would be less potential cost saving as the pathway would already be closer to optimality (Fig. 2).

Proportionately upscaling the £2.1m saving to the national level therefore requires sufficient similarity to this ‘starting place’. Using the same centrally provided data as for the studied healthcare system, and applying the same definitions, gives an average 1489 acute beds occupied by community DTOC patients in all of England (see the ESM). With the approximate 56 million England population, this corresponds to an average 26.6 acute DTOC beds occupied per 1 million population. This compares favourably to the above-mentioned 26.3 (for the 1 million BNSSG population), suggesting that local circumstances are not unrepresentative of those nationally.

Thus, multiplying through the £2.1m by 56 provides a £117m estimate of the potential annual cost saving in England. As calculated earlier, this would be achieved through a 3% expansion of post-acute community capacity, which would reduce acute DTOCs to the 12% of current levels that can be regarded as economically essential. This additional capacity would cost £45m, versus the £162m cost saving in the acute setting. Note that the net cost saving relates only to improvements in NHS-funded community care and does not represent the full cost saving potential that may be possible if improvements were also made to local authority social care (which, as mentioned in the Introduction section, is accountable for 28% of DTOCs).

## Discussion

Although a stated aim for many NHS systems [6–10], this study finds that seeking to ‘eliminate’ DTOCs is likely a false economy: simply put, the additional community costs that would be required to achieve this aim are greater than the possible acute cost saving. We find that a large proportion (88%) of the average 26.6 acute DTOC beds per million population in England’s NHS can be attributed to inefficient use of resources. This indicates substantial scope for improving the efficiency of complex discharge pathways, through significantly reducing, but not eradicating, acute DTOCs. This study makes an important contribution to the literature: while others have linked community resources to acute DTOCs, there has been a deficit of work considering optimal resource allocation.

In terms of the £117m cost saving estimated here, the literature provides few examples for direct comparison. The National Audit Office has previously estimated a gross cost of £820m for acute DTOCs in England [25]. However, this figure, calculated in 2016, accounts for delays caused by local authority-funded social care as well as those attributable to NHS-funded community services (the scope of the current study). It also includes a 2.7-fold uplift (of fairly nebulous provenance) applied to the DTOC volumes “to account for patients no longer benefiting from acute care, excluded from the definition of delayed transfers of care”. While the non-acute costs required to achieve the removal of acute DTOCs are estimated, there is no consideration to the variability that realistically exists and which prohibits the kind of simplistic analysis based on DTOC ‘elimination’. In another study, Allan et al. [2] use regression modelling to consider the effect of local authority-funded social care spending on acute DTOCs in England. They find that “home care supply significantly reduced DTOCs” and that increases to social care capacity over the period from 2011 to 2016 led to overall cost savings of between £73m and £274m.

With respect to practical implications, the findings of this study suggest that aiming for ‘zero DTOC’ targets is misguided. Instead, an assessment should be made based on the particular characteristics and conditions of the considered hospital or healthcare system. This would avoid the larger community capacity requirements associated with ‘eliminating’ DTOCs, which is particularly important given the current and longer-term workforce recruitment and retention issues in the healthcare sector [26]. Managers can also lessen required capacity through reducing inherent variability in the system. Here, we demonstrate the effect of this on community capacity through hypothetically adjusting length of stay variability. Efforts could also be made to reduce the variability in ‘arrivals’, e.g. through ‘smoothing’ the scheduling of elective care procedures for patients expected to require continuing post-acute care [27]. Another option, not explored in this current study, is to optimise the real-time procurement of short-term agency capacity, to coincide with community demand peaks as they occur. This would involve balancing the higher costs of agency resources against the value they bring in reducing acute DTOCs and increasing community capacity utilisation (such an approach has previously been considered in the critical care setting [28]).

In terms of technical limitations, it should be acknowledged that one mathematical formula presented in the Methods section (Eq. 3) is an approximation and not an exact result, although it has been found to be “usually an excellent approximation” [29]. It is also worth acknowledging that data from a variety of sources are used for model calibration, since no single source was available. While such data limitations are a recognised reality of working across various healthcare settings [30], and especially community care [31], they would not be expected to materially affect the overall conclusions of this study (although they may have some limited effect on the particular results; for instance, if more patients are routed to cheaper home-based rather than bed-based community care, then cost optimality would result from increasing community capacity by some amount, while further penalising acute DTOCs).

With respect to the scope of this study, it should be noted that our analysis covers only DTOCs attributable to NHS-funded community care, and not those related to disputes, patient choice, and insufficiency of local authority-funded social care capacity (the latter of which could be considered using a similar approach to that taken here). In terms of financial costs, the scope is restricted only to those directly associated with acute and community service provision, and not those related to other parts of the patient pathway. Upstream, DTOCs associate with increased acute bed occupancy, which can lead to emergency department overcrowding and, in turn, ambulance offload delays, all of which require additional capacity and cost to address [32, 33]. DTOCs may also lead to a greater risk of hospital-acquired infection, again requiring additional resources to address. Downstream, the effect of DTOCs on functional and cognitive decline (so-called ‘deconditioning’) could mean a costlier long-term care placement when cheaper home-based care could otherwise have sufficed [34]. The effect of both these upstream and downstream effects would further penalise acute DTOCs, somewhat lowering the tolerated ‘optimal’ levels reported in the Results section; so too would consideration to the patient’s own preference for timely transfer upon discharge readiness, although further thought would be required as to how this ‘quality of life’ aspect can be accounted for in equations otherwise denominated by financial cost alone.

## Conclusions

The additional community costs required to ‘eliminate’ DTOCs are greater than the possible acute cost saving. Some amount of DTOCs should therefore be regarded as economically essential, and the use of ‘zero DTOC’ targets should be discouraged. Secondarily, this study finds that Operational Research methods such as Queueing Theory can be advantageous to investigating policy matters in this domain.

## Supplementary Information

Below is the link to the electronic supplementary material.

Supplementary file1 (DOCX 55 kb)

## Declarations

### Data availability

All data generated or analysed during this study are included in this published article.

### Code availability

The code and data used are available from the corresponding author upon reasonable request.
