# Correction to: The False Economy of Seeking to Eliminate Delayed Transfers of Care: Some Lessons from Queueing Theory

**DOI:** 10.1007/s40258-023-00821-9

**Published:** 2023-07-03

**Authors:** Richard M. Wood, Alison L. Harper, Zehra Onen-Dumlu, Paul G. Forte, Martin Pitt, Christos Vasilakis

**Affiliations:** 1 UK National Health Service (BNSSG ICB), NHS Bristol, North Somerset and South Gloucestershire Integrated Care Board, 360 Bristol, Marlborough St, Bristol, BS1 3NX UK; 2 School of Management, University of Bath, Bath, UK; 3 Medical School, University of Exeter, Exeter, UK; 4 Health Data Research UK, South West Better Care Partnership, Bristol, UK

**Correction to: Applied Health Economics and Health Policy (2023) 21:243–251** 10.1007/s40258-022-00777-2

This article was originally published under a CC BY-NC 4.0 license, but has now been made available under a Creative Commons Attribution 4.0 International License (https://creativecommons.org/licenses/by/4.0/), which permits use, sharing, adaptation, distribution and reproduction in any medium or format, as long as you give appropriate credit to the original author(s) and the source, provide a link to the Creative Commons licence, and indicate if changes were made. The PDF and HTML versions of the paper have been modified accordingly.

The original article has been corrected.

