# Supplementary material for: The False Economy of Seeking to Eliminate Delayed Transfers of Care: Some Lessons from Queueing Theory
Source: Appl Health Econ Health Policy. 2022 Dec 18;21(2):243–51. doi: 10.1007/s40258-022-00777-2 (PMC9760184; doi:10.1007/s40258-022-00777-2)
Supplement: Supplementary file 1 — Supplementary file1 (DOCX 55 kb) [file 40258_2022_777_MOESM1_ESM.docx]

**Supplementary Information**

Applied Health Economics and Health Policy

The false economy of seeking to eliminate delayed transfers of care: some lessons from queueing theory

Richard M Wood ^a,b,d^

Alison L Harper ^c,d^

Zehra Onen-Dumlu ^b,d^

Paul G Forte ^a,d^

Martin Pitt ^c,d^

Christos Vasilakis ^b,d^

^a^ UK National Health Service (BNSSG CCG), Bristol, UK.

^b^ School of Management, University of Bath, Bath, UK.

^c^ Medical School, University of Exeter, Exeter, Exeter, UK.

^d^ Health Data Research UK South West Better Care Partnership, UK.

Correspondence: Dr Richard M Wood; richard.wood16@nhs.net.

Funding: This work was supported by Health Data Research UK, which is funded by the UK Medical Research Council, Engineering and Physical Sciences Research Council, Economic and Social Research Council, National Institute for Health Research, Chief Scientist Office of the Scottish Government Health and Social Care Directorates, Health and Social Care Research and Development Division (Welsh Government), Public Health Agency (South Western Ireland), British Heart Foundation and Wellcome (award number CFC0129).

Conflict of interest: All authors declare that they have no conflict of interest.

***Parameter calibration for the studied healthcare system (Methods; Data and calibration)***

The average daily community cost across both home-based and bedded care was estimated as follows.

Time-limited ‘step down’ community services in the studied healthcare system follow the Discharge To Assess (D2A) model consisting of the three pathways: P1 (home with care visits), P2 (bedded care), and P3 (more complex bedded care). From local data, 59% of acute patients requiring community care go to P1, 22% to P2, and 19% to P3. Unit costs (per patient per day) are estimated at £125 for P1 [1], £150 for P2 [2], and £164 for P3 (local data). Note that data limitations at the studied healthcare system prevented the use of local data for estimating all such parameters. Combining the unit costs for the community service as a whole yields the community unit cost estimate, i.e. 0.59×£125 + 0.22×£150 + 0.19×£164 = £138. With the acute unit cost (α) equalling £346, this gives β=0.399.

The coefficient of variation (V) for community service length of stay (LOS) was calculated as 0.965 from local data (for all D2A pathways P1-3). Community service capacity (c) was calculated as the addition of the capacities of the individual D2A pathways. With P1 having a capacity for 182 concurrent patients, P2 for 177 patients, and P3 for 172 patients, this meant a total community service capacity of 531.

Finally, traffic intensity ($\rho$) was estimated based upon the deduced average amount of acute beds occupied by community DTOC patients at the studied healthcare system. Publicly available data [3] quantifies the annual amount of acute DTOC bed days lost due to community service unavailability at 9616 for the studied healthcare system. Thus, on an average day, there were 26.3 patients awaiting discharge to the community service. Putting this into Eqn. (4) and solving for $\rho$ gives the traffic intensity estimate of 0.98.

***Calculation of average acute beds occupied by community DTOC patients in all of England (Results; Cost saving potential for England)***

Using the same data as above [3], but not restricting to the studied healthcare system, gives an England total annual amount of acute DTOC bed days lost due to NHS funded community service unavailability of 543,529. Thus, on an average day, there were 1489 patients awaiting discharge to the community service.

***References***

[1] National Health Service (NHS) Improvement. 2017/18 Reference Costs. 2019. <https://webarchive.nationalarchives.gov.uk/ukgwa/20200501111106/https://improvement.nhs.uk/resources/reference-costs/>.

[2] NAIC. National Audit of Intermediate Care: Summary Report England 2017. 2017. <https://s3.eu-west-2.amazonaws.com/nhsbn-static/NAIC+(Providers)/2017/NAIC+England+Summary+Report+-+upload+2.pdf>.

[3] National Health Service (NHS) England. Delayed Transfers of Care Data 2019-20. 2020. <https://www.england.nhs.uk/statistics/statistical-work-areas/delayed-transfers-of-care/delayed-transfers-of-care-data-2019-20/>.
